# Update on stomata development and action under abiotic stress

**DOI:** 10.3389/fpls.2023.1270180

**Published:** 2023-10-02

**Authors:** Hubert Matkowski, Agata Daszkowska-Golec

**Affiliations:** Institute of Biology, Biotechnology and Environmental Protection, Faculty of Natural Sciences, University of Silesia in Katowice, Katowice, Poland

**Keywords:** stomata, stress, plants, abiotic stress, stomata development, climate change

## Abstract

Stomata, key gatekeepers of plant hydration, have long been known to play a pivotal role in mitigating the impacts of abiotic stressors. However, the complex molecular mechanisms underscoring this role remain unresolved fully and continue to be the subject of research. In the context of water-use efficiency (WUE), a key indicator of a plant’s ability to conserve water, this aspect links intrinsically with stomatal behavior. Given the pivotal role of stomata in modulating water loss, it can be argued that the complex mechanisms governing stomatal development and function will significantly influence a plant’s WUE under different abiotic stress conditions. Addressing these calls for a concerted effort to strengthen plant adaptability through advanced, targeted research. In this vein, recent studies have illuminated how specific stressors trigger alterations in gene expression, orchestrating changes in stomatal pattern, structure, and opening. This reveals a complex interplay between stress stimuli and regulatory sequences of essential genes implicated in stomatal development, such as *MUTE*, *SPCH*, and *FAMA*. This review synthesizes current discoveries on the molecular foundations of stomatal development and behavior in various stress conditions and their implications for WUE. It highlights the imperative for continued exploration, as understanding and leveraging these mechanisms guarantee enhanced plant resilience amid an ever-changing climatic landscape.

## Introduction

Water deficiency, which results from rising global temperatures, is one of the main factors limiting plant productivity. It significantly reduces the yield of plants, thus causing food shortages and leading to worldwide food insecurity problems (Ferguson, 2019). Plants have developed many defense mechanisms to cope with unfavorable conditions. Under abiotic stress, the stomatal reaction often occurs as one of the first within a few seconds or a few minutes after the stressor appearance. Such a rapid response highlights the vital role of stomata in avoiding abiotic stresses in plants (Ferguson, 2019; Kollist et al., 2019). Here, we reviewed recent findings regarding the molecular basis of stomata development and action under abiotic stresses, including crop species (**Table 1**).

**Table 1:** Factors affecting the functioning and development of stomata in cultivated plant species.

Plant species	Abiotic stress	Factor	Description	Reference
*Daucus carota*	Drought	DcABF3	The overexpression of *ABA Binding Factor 3* from *Daucus carota* (*DcABF3)* results in increased expression of *MUTE, FAMA* and SPEECHLESS (*SPCH)* influencing the stomatal density (SD)	(Wang et al., 2021)
*Zea Mays*	Drought	ZmNAC49	The overexpression of *ZmNAC49* increases the expression of *TOO MANY MOUTHS* (*TMM)*, *STOMATAL DENSITY AND DISTRIBUTION 1* (*SDD1)* and *FAMA* and decreases *MUTE* resulting in a decrease in SD compared to WT. Within the *MUTE* promoter, a specific sequence has been identified to which NAC family TFs bind.	(Xiang et al., 2021)
*Hordeum vulgare*	Drought	miRNA393 (*HvMIR393*)	Overexpression of *miRNA393* causes a reduction in Guard Cells (GC) size while increasing stomatal density. The mutant with suppressed expression of *miRNA393* has the opposite phenotype and shows increased expression of *SPCH* and *MUTE*.	(Yuan et al., 2019)
*Oryza sativa*	Saltinity and drought	*HDA704*	Overexpression of histone deacetylase *HDA704* in drought conditions results in a higher number of fully closed stomata, while in the knockout mutant the number of fully closed stomata is lower than in WT.	(Zhao et al., 2021)
*Oryza sativa*	Salinity	*OsVPE3*	Inhibition of *VACUOLAR PROCESSING ENZYME 3 (OsVPE3)* function results in a reduction in stomata size under salt stress and a reduction in the expression of *OsTMM*, *OsSPCH1* and *OsMUTE.*	(Lu et al., 2016)
*Arabidopsis thaliana*	Drought	*NDT1 (At2g47490), NDT2 (At1g25380)*	Downregulation of *NAD+ TRANSPORTER 1 (NDT1)* and *NAD+ TRANSPORTER 2 (NDT2)* results in a decrease in stomata number. It increases the expression of *TMM, SSD1, EPIDERMAL PATTERNING FACTOR 1 (EPF1)* and *EPIDERMAL PATTERNING FACTOR 2 (EPF2)* and reduces the expression of *SPCH* and *MUTE*, which reduces the meristemoid cell index.	(Feitosa-Araujo et al., 2020)
*Arabidopsis thaliana*	Drought	*AtBG1 (AT1G52400); vat1 (AT2G18790)*	The *AtBG1/vat1* (BETA GLUCOSIDASE 18 and PHYTOCHROME B) double mutant is characterized by a larger stomatal aperture and reduced expression of *SPCH* and *MUTE*	(Allen et al., 2019)
*Arabidopsis thaliana*	Drought	SnRK2s kinases (AT3G50500, AT5G66880, AT4G33950)	Mutants in the genes encoding the SnRK2.2 and SnRK2.3 kinases are characterized by an increase in the Stomatal Index and in the double mutant snrk2.2/snrk2.3 this increase is at a higher level than in single mutants. In the snrk2.2/snrk2.3/snrk2.6 triple mutant no inhibitory effect of ABA on stomatal index (SI) is observed.	(Yang et al., 2022)
*Arabidopsis thaliana*	Drought	*KIN10 kinase (AT3G01090)*	Increased expression of *KIN10* results in an increase in SI, lower expression of *KIN10* causes a decrease in SI by interacting with the *SPCH* gene.	(Han et al., 2020)
*Arabidopsis thaliana*	Drought	ERECTA *( AT2G26330)*	By interacting with E2Fa-transcription factor, it modulates the expression of genes related to the cell cycle, causing premature entry of cells into the endoreduplication cycle, affecting their larger sizes	(Guo et al., 2019)
*Arabidopsis thaliana*	Drought	NADPH oxidases (*ATRBOHF/AtRBOHD*)	A mutation in the *ARABIDOPSIS THALIANA RESPIRATORY BURST OXIDASE HOMOLOG F (AtRBOHF)* gene reduces ABA-associated stomatal closure, a double mutation in the *AtRBOHF/AtRBOHD* genes results in a stronger response, and a mutation in the *ARABIDOPSIS THALIANA RESPIRATORY BURST OXIDASE HOMOLOG D (AtRBOHD*) gene causes no response to ABA-associated stomatal closure	(Sierla et al., 2016; Ma and Bai, 2021)
*Arabidopsis thaliana*	Heat	*PIF4* *(AT2G43010)*	The increase in the expression of *PHYTOCHROME INTERACTING FACTOR 4 (PIF4*) which is expressed, among others, in in stomatal precursors during heat stress causes a decrease in *SPCH* expression, while loss-of-function *pif4* and *pif4-2* mutants show an increase in *SPCH* expression	(Lau et al., 2018)
*Arabidopsis thaliana*	Light	*ERA1 (AT5G40280)*	Mutant *era1-2* carrying a mutation in the *ENHANCED RESPONSE TO ABA 1 (ERA1)* gene, which is involved in blue light-dependent stomatal opening, causes a decrease in stomatal conductance compared to WT	(Jalakas et al., 2017)
*Arabidopsis thaliana*	Light	*HY5 (AT5G11260)*	Overexpression of *ELONGATED HYPOCOTYL 5* (*HY5)* causes an increase in SI and SD in proportion to light intensity by interacting with *SPCH*, while the *hy5*-*215* mutation causes a decrease in SI and SD compared to WT.	(Wang et al., 2021)
*Arabidopsis thaliana*	Heavy Metal	*LCD (AT3G62130)*	Mutation in the *L-CYSTEINE DESULFHYDRASE (LCD)* gene reduces endogenous H_2_S. Reducing the level of hydrogen sulfide causes disturbances in the signalling associated with JA corresponding to, among others, for the development of stomata by regulating the activity of transcription factors MYC2, MYC3 and MYC4, which affect the suppression of the expression of *SPCH*, *MUTE*, *FAMA* and *TMM*. The mutant in the *LCD* gene is characterized by a higher density of stomata than WT	(Deng et al., 2020)

## Genetic insights into stomata development in monocots and dicots

Stomata are microscopic pores in the epidermis of the above-ground parts of plants surrounded by two cells called guard cells (GC) (Pillitteri and Torii, 2012). The turgor of the guard cells drives the opening and closing of the stomata. This regulation enables the uptake of CO_2_ necessary for photosynthesis, and the discharge of O_2_, additionally allows the regulation of internal temperature through transpiration, i.e., evaporation of H_2_O, thus maintaining the gas balance in the plant (Chater et al., 2017; Zoulias et al., 2018).

**Figure 1:**
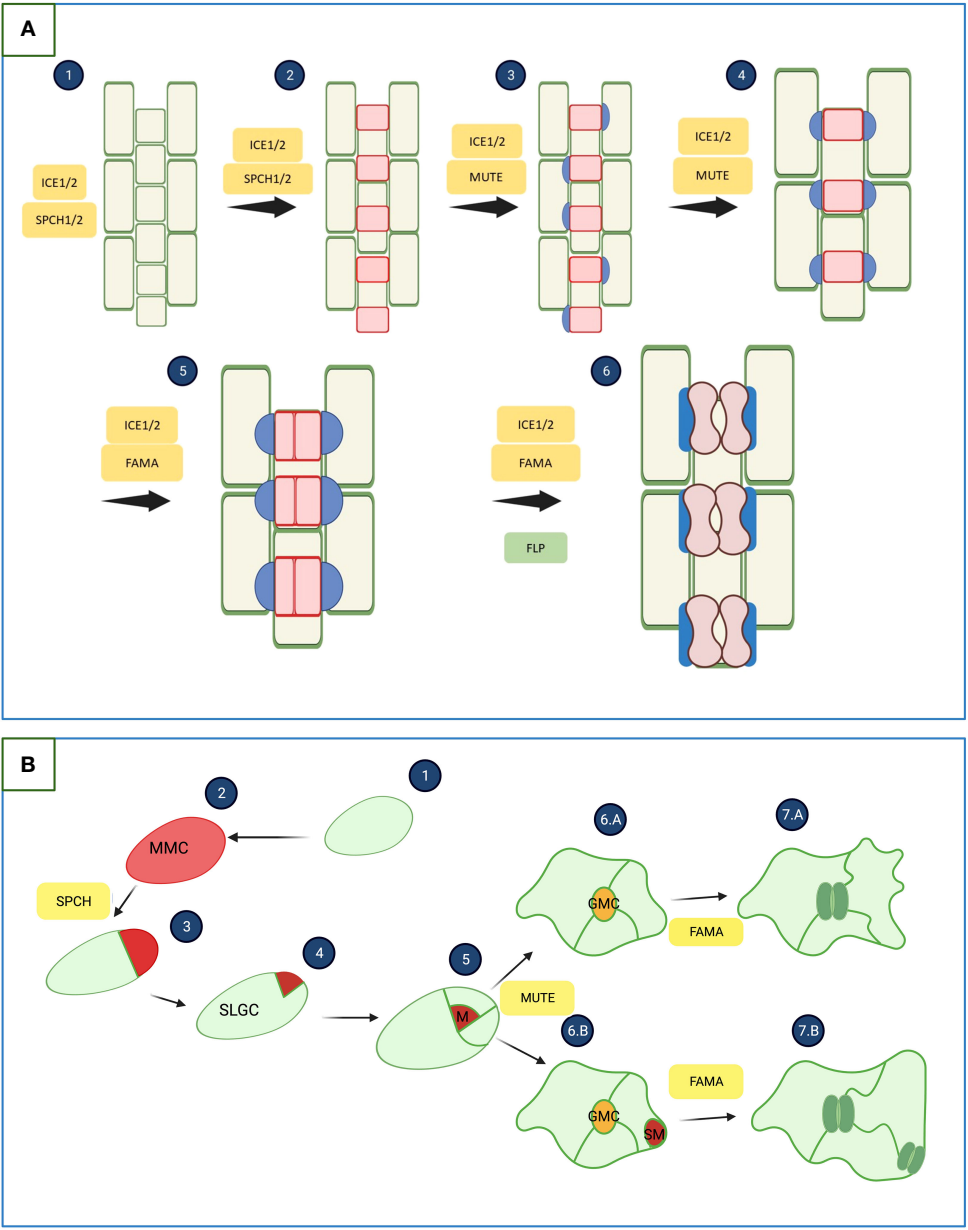
Development of stomata in monocotyledonous and dicotyledonous plants. **(A)** Genetic basis of stomata development in monocotyledonous plants. 1. Selection of stomatal lineage cells. 2. Asymmetric divisions generating guard mother cells (GMC). 3. GMCs expand, which induces the formation of subsidiary mother cells (SMC). 4. SMC maturation. 5. GMCs divide symmetrically to form two guard cells. 6. Elongation and maturation of the GMC, finally forming two mature guard cells. SPEECHLESS 1/2 (SPCH1/2) proteins are responsible for stomatal identity acquisition and first division. MUTE promotes the formation of subsidiary cells. The symmetrical division and maturation of Guard Mother Cells is promoted by FAMA and FLP. INDUCER OF CBF EXPRESSION 1/2 (ICE1/2) forms heterodimers with SPCH1/2, MUTE and FAMA (Hepworth et al., 2018; Wei et al., 2020). **(B)** Genetic basis of stomata development in dicotyledonous plants. (1), (2) - The protodermal cell undergoes transformation into a meristemoid mother cell (MMC), (3), (4), (5) - the MMC undergoes a series of asymmetric divisions to form a meristemoid (M) and stomatal-lineage ground cell (SLGC) (Controlled by *SPECHLESS (SPCH)*). SLGCs may divide into pavement cells (6.A) or differentiate into satellite meristemoid (SM) (Controlled by *MUTE*) (6.B). The meristemoids divide asymmetrically and eventually become the guard mother cell (GMC). The GMC divides once symmetrically to form two guard cells (GC) of equal size (7.A), (7.B) (Controlled by *FAMA*) (Lau and Bergmann, 2012; Chen et al., 2020). The illustration was created using BioRender (www.biorender.com).

Considering the pattern of the epidermis of monocotyledonous and dicotyledonous plants, one can notice differences in the structure and arrangement of the stomata. One of the differences between monocots and dicots stomata is the shape of the guard cells (GC). In monocots, GC is dumbbell-shaped, while in dicots kidney-shaped (**Figure 1**). Another notable difference between monocots and dicots is the stomatal pattern. The epidermis of monocotyledonous plants is characterized by the arrangement of stomata in parallel rows (**Figure 1A**). It is postulated that this arrangement is related to the venation of the leaves, which in monocots are arranged parallel. In addition, GC in monocots are surrounded by a pair of subsidiary cells that come from adjacent, non-linearly related cells. In dicotyledonous, it is also postulated that stomatal arrangement is associated with the structure of leaf venation, which is less organized, translating into a more chaotic pattern of stomatal arrangement (**Figure 1B**). One of the main principles of epidermal pattern formation in dicots is that there must be at least one-cell space between the stomata (Hepworth et al., 2018; Conklin et al., 2019; Rudall, 2023).

In dicotyledonous plants, stomata development is conditioned by a series of symmetrical and asymmetric divisions of protodermal cells called meristemoid mother cells (MMC). It starts from the asymmetric division of the MMC, resulting in a small meristemoid and a stomatal lineage ground cell (SLGC). The meristemoid cell (MMC) can undergo up to three asymmetric divisions spiraling inwards. A late meristemoid then forms, surrounded by SLGC. The late meristemoid then differentiates into a Guard Mother Cell (GMC), which undergoes one symmetrical division to form a pair of guard cells (**Figure 1B**). These divisions occur both on the leaf’s adaxial and abaxial sides. For example, in *Arabidopsis thaliana*, most of the stomata are formed on the abaxial side of the leaf (Martin and Glover, 2007; Qi and Torii, 2018; Zoulias et al., 2018). It is postulated that the arrangement of the stomata on the underside of the leaf is related to their function, which prevents the loss of water from the plant. Sunlight heats the abaxial side less due to its limited access, which prevents water evaporation from the plant through the stomata (Martin and Glover, 2007). The transcription factors of the basic helix–loop–helix (bHLH) family: SPEECHLESS (SPCH), MUTE, and FAMA, together with the accompanying proteins SCREAM1 (SCRM1) and SCREAM2 (SCRM2), are responsible for controlling above-mentioned series of divisions. SPCH is necessary to induce asymmetric division, while MUTE interrupts these divisions and promotes symmetric ones (Pillitteri and Torii, 2012; Qi and Torii, 2018). These five transcription factors can be characterized as the central genes controlling the morphogenesis of the stomata. However, other genes such as *FOUR LIPS, YODA*, and *ERECTA* are also involved in this process, the cooperation of which enables the entire process of stomatal formation (Martin and Glover, 2007; Pillitteri and Torii, 2012; Qi and Torii, 2018). In monocots, the same genes are involved in stomata development and differentiation as in dicots, but their functioning is different (Chater et al., 2017; Hepworth et al., 2018). Apart from the shape GCs, the main difference is that stomata are surrounded by additional Subsidiary Cells (SC). The stomatal development of monocotyledonous plants can be visualized based on *Hordeum vulgare* and divided into 6 stages (**Figure 1A**): (1) at the base of the leaf, potential precursor cells proliferate in specific clusters; (2) undifferentiated cells are pushed up the leaf blade, and alternating cells enter the path of stomatal differentiation through the first asymmetric division, resulting in the GMC and a larger sister cell; (3) cells from the clusters surrounding the newly formed GMC divide asymmetrically to form subsidiary mother cells (SMC); (4) the GMCs increase in size and during this process two emerging SCs surround them; (5) final division of GMCs, resulting in two immature GCs; (6) the stomatal complex matures, resulting in stomata surrounded by two dumbbell-shaped GCs, which is surrounded by two SCs (Hepworth et al., 2018).

Studies on the functions of the *SPCH*, *SCRM*, and *ICE1* (I*NDUCER OF CBF EXPRESSION1*) genes have shown differences between monocots and dicots. In monocots, speciation of *ICE1* and *SCRM2* has been shown to occur, while in dicots such as *A. thaliana*, they appear redundant. In addition, SPCH is duplicated and neofunctionalized in monocots, which may indicate the occurrence of new evolutionary pressures. The critical issue is the acquisition of the mobility of the MUTE protein, thanks to which the function in modeling subsidiary cells was obtained (Chater et al., 2017; Rudall et al., 2017). Monocotyledonous plants usually lack SPCH, MUTE, and FAMA protein homologs, while dicotyledonous plants have been shown to have as many as 2-3 groups of homologues with highly conserved genetic similarity. Studies on the structure of the SPCH protein between monocots and dicots showed that the bHLH domain is highly conserved in both groups. However, different folding of the peptide and the structure of other domains were found. A particular difference was shown in the target domain of MAPK, which indicates a different sensitivity to *SPCH* phosphorylation by MAPK (Song et al., 2023).

## Stomatal development and function under drought

Drought is a hazardous phenomenon that tremendously impacts plants functioning. It triggers physiological and biochemical reactions aimed at mitigating its effects and enabling the plant to survive drought conditions (Pirasteh-Anosheh et al., 2016). Root system growth and development are promoted to increase water availability, allowing water uptake from deeper soil layers. This is also done at the expense of the above-ground parts of plants, where, due to insufficient water, the leaf cells lose their turgor, resulting in wilt. Some plants (i.e. *Macroptilium atropurpureum*) can control the movement of the leaf blades by phototropism, aligning the leaf parallel to the incident light rays, reducing water loss in the plant (Fang and Xiong, 2015). Drought also leads to disturbances in the photosynthesis process, caused by insufficient CO_2_ entering the plant due to the closing of the stomata (Xu et al., 2010; Zargar et al., 2017). Stomata play a role in avoiding drought by limiting transpiration, which translates into lower water loss, so when there is a water shortage in the soil, the stomata are closed (Tombesi et al., 2015).

Environmental signals significantly affect the aperture of stomata, thus modulating the stomata conductance (g_s_), affecting the ability to exchange gas between the plant and the environment. Changes in g_s_ can last from a few minutes to several hours. A slow increase in g_s_ may limit the rate of photosynthesis, while a slight decrease in g_s_ negatively affects Water Use Efficiency (WUE) (Lawson and Vialet-Chabrand, 2019). WUE is defined as the amount of fixed carbon as biomass in the plant per water unit used (Hatfield and Dold, 2019). Changes in g_s_ limit water transpiration and optimize CO_2_ uptake, thereby modulating the photosynthesis process (Bertolino et al., 2019). In unfavorable environmental conditions, the plant modifies the arrangement and size of stomata, affecting the optimization of g_s_. For example, in drought conditions in *A. thaliana*, the stomata’s size decreases, which contributes to water savings and better WUE (Doheny-Adams et al., 2012; Bertolino et al., 2019). In cereals, where guard cells are dumbbell-shaped, it has been observed that reducing their size causes them to close by increasing the ion flow between cells, which allows faster changes in g_s_ and better affects WUE (Bertolino et al., 2019).

Two *Zea mays* varieties, XY335, which is a hybrid of line PH6WC x PH4WC, introduced in the US, and ZD958, which is a hybrid of line Zheng58 × Chang7-2 and developed in China, were tested in the field to investigate the effect of combined drought and salinity stresses on stomatal conductance and WUE. Mild drought and salt stress caused a decrease in g_s_, photosynthesis rate (A), and an increase in WUE with the rate of plant development, with an increase in WUE observed for various parts of plants: aboveground biomass water use efficiency (WUE_b_), grain yield water use efficiency (WUE_g_) (Liao et al., 2022). Whether changes in stomata distribution pattern result in better utilization in drought conditions was checked in rice. For this purpose, mutants of high stomatal density (HD), low stomatal density (LD), small stomata (SS), and large stomata (LS) were identified. It was shown that HD plants had larger g_s_ than the other mutants, with HD plants also having larger stomatal sizes. However, the results suggest that stomatal density, not stomatal size, contributes to increased g_s_ (Pitaloka et al., 2022). The response of these four mutants to drought was also tested. In LD plants under drought conditions, biomass and yield are higher than in other lines, these plants were also characterized by better WUE, suggesting that lower stomatal density in rice improves drought adaptation (Pitaloka et al., 2022).

Plants have developed mechanisms to avoid drought stress, in which abscisic acid (ABA) plays a critical role (Liu et al., 2018). Under stress, ABA-related signalling pathways regulate the transcriptional activity of stress-avoiding genes, thereby regulating physiological processes, including the stomatal closure (Collin et al., 2020). ABA is one of the primary regulators of stress response in plants. Its level increases under stress conditions. In addition, it regulates many growth and development processes (Daszkowska-Golec, 2016). ABA receptors PYRABACTIN RESISTANCE1 (PYR/PYL/RCAR)/PYR1-LIKE (PYL)/REGULATORY COMPONENTS OF ABA RECEPTORS (RCAR), interact with Protein Phosphatases 2C (PP2Cs), which are negative regulators of ABA signalling. Receptors bind to PP2Cs and inactivate them. In turn, activation of other PP2C targets, such as SNF1-related protein kinases (SnRK2) and S-type anion channels (SLAC), occurs. Activation of the slow-type anion channel (SLAC1) allows ions to flow across the guard cell membrane causing the stomata to close. ABA in *A. thaliana* promotes the activity of SnRK2.6 kinases, which is OPEN STOMATA 1 (OST1) kinase. This kinase phosphorylates the transcription factors ABA BINDING FACTOR2 (ABF2) and ABA BINDING FACTOR3 (ABF3), which bind to ABA-responsive elements (ABRE), affecting the expression of ABA-dependent genes. ABF factors are also responsible for promoting stomatal closure. In addition, the *ost1* mutant is characterized by greater sensitivity to drought and increased water loss due to the permanently open stomata (Lim et al., 2015).

Studies on one of the ABF3 from *Daucus carota*, a nuclear protein, showed that it is involved in the drought response because its expression increased in water scarcity conditions. Transgenic *A. thaliana* plants overexpressing *DcABF3* have shown that overexpression of this gene increases the expression of *FAMA, MUTE, SPCH, SCRM1*, and *SCRM2*, resulting in higher stomatal density. In addition, germination of WT plants and lines with overexpression of *DcABF3* in the presence of exogenous abscisic acid showed that overexpression of *DcABF3* resulted in reduced sensitivity to ABA, WT showed a lower germination rate than overexpression line (Wang et al., 2021).

Recent studies on *A.thaliana* have shown that nicotinamide adenine dinucleotide (NAD) is involved in the negative regulation of stomatal formation. This mechanism is related to the enhanced ABA signalling (Feitosa-Araujo et al., 2020). NAD^+^ takes place in oxidation and reduction reactions as a cofactor for energy transfer and also acts as an essential signalling molecule, including stress-related signalling (Gakière et al., 2018). A publicly available transcriptomic data meta-analysis showed that expression of NAD^+^ biosynthetic genes and NAD^+^ carrier genes *NAD^+^ Transporter 1* and *NAD^+^ Transporter 2* (*NDT1* and *NDT2*) is strongly induced in stomatal guard cells compared to other leaf cells. Insertional *A.thaliana* mutants in *NDT1* and *NDT2* genes were used to test the role of these genes in stomatal development. Reduced expression of NAD^+^ carrier genes resulted in a decrease in stomatal numbers compared to WT plants. The number and index of meristemoids also decreased in the mutants while increasing the number of GMCs. It was also examined how a change in NAD^+^ distribution affects the expression of key regulators of stomatal development. *TOO MANY MOUTHS* (*TMM)*, *STOMATAL DENSITY AND DISTRIBUTION 1* (*SDD1)*, *EPIDERMAL PATTERNING FACTOR 1 (EPF1)* or *EPIDERMAL PATTERNING FACTOR 2* (*EPF2)* were upregulated in mutants in the *NDT* genes. In contrast, the expression of genes promoting stomata development, such as *SPCH* and *MUTE*, was inhibited in both mutants. It was also checked whether treatment with exogenous NAD^+^ would affect stomatal development. For this purpose, plants were grown with 1.5 mM NAD^+^. It was found that treatment with exogenous NAD^+^ resulted in a higher NAD^+^ content compared to control conditions. Using a fluorescent sensor of the cytosolic and nuclear NAD redox state, peredox-mCherry has proven that exogenous NAD^+^ can enter cells and disrupt NAD^+^ homeostasis. A comparison of the effect of exogenous NAD^+^ on mutants in the NAD+ transporter genes and WT showed that NAD^+^ treatment reduced Stomatal Index (SI) in the WT. In contrast, no such effect was observed in mutants. NAD^+^ has also been shown to increase the expression of the *RESPONSIVE TO DESICCATION 29B* (*RD29B*) gene, demonstrating the potential role of the ABA signalling pathway as an intermediary in controlling stomatal development via NAD^+^. Using the ABA-sensitive fluorescent protein-based FRET sensor ABA-leon2.1, it was proved that in the case of exogenous NAD^+^ treatment, the concentrations of ABA in the cytosol and cell nucleus are higher compared to the control (Feitosa-Araujo et al., 2020).

Since ABA plays a vital role in the formation and functioning of stomata, plants need to maintain homeostasis of endogenous ABA through its synthesis and degradation reactions to ensure proper perception of the signalling pathways associated with it (Pantin et al., 2013; Xu et al., 2013; Chater et al., 2014). ABA homeostasis is mediated by *β-GLUCOSIDASE 1* (*AtBG1*), which is involved in cleaving the inactive ABA conjugate (ABA-glucose ester), thereby releasing ABA, which can then accumulate in the cytoplasm (Harris and Ondzighi-Assoume, 2017). A mutation in the *AtBG1* (*atbg1*) gene in *A. thaliana* negatively affects drought tolerance by increasing stomatal density in plants with an *atbg1* mutation. Loss function in the *ATBG1* gene impacted on increased expression of genes controlling the development of stomata such as *SPCH, FAMA, MUTE*. It was also tested how an additional *vat1* mutation would affect the phenotype of the *atbg1* mutant. The *vat1* mutant carries mutations in the phytochrome B (PHYB) gene, and it was isolated by screening the ethyl methanesulfonate (EMS) mutant *atbg1* population. It was named *trait variant atbg1 1 (vat1)*, identified as a novel phytochrome B (PHYB) allele responsible for the inability to respond to red light. The identified point mutation restored drought tolerance to the *atbg1* mutant. Analysis of the single mutant *vat1* also showed a slight but significant increase in drought tolerance compared to WT. A change in stomatal density has been postulated to be a mechanism for drought tolerance in the *vat1* mutant. The *vat1* mutant and the double mutant *atbg1/vat1* did not show statistically significant differences compared to the WT. It was also checked how these mutations affect the stomata aperture, which slightly increased in the *vat1* mutant compared to the WT, while a higher stomatal aperture characterized the *atbg1/vat1* double mutant. In both the *vat1* mutant and the *atbg1/vat1* double mutant, the expression of *SPCH* and *MUTE* genes was reduced (Allen et al., 2019).

**Figure 2:**
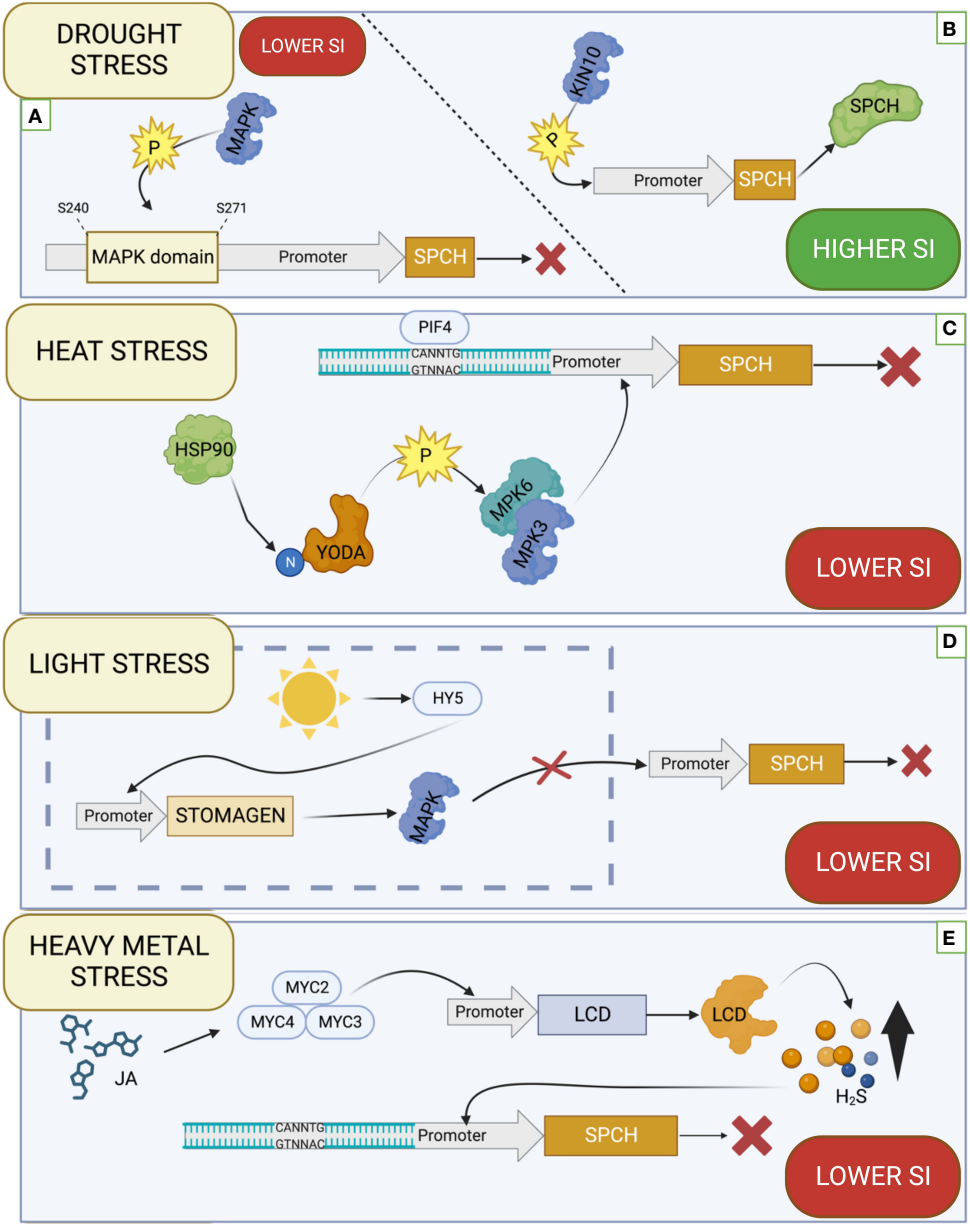
Effect of various factors on the function of the *SPEECHLESS* (*SPCH*). **(A)** SNF1-related protein kinases 2 (SnRK2) kinase phosphorylate targets within the MAPK domain of the *SPCH* promoter, inhibiting its expression. **(B)** KIN10 phosphorylates the SPCH protein promoting its functioning. **(C)** HEAT SHOCK PROTEIN 90 (HSP90) affects the N-terminal regulatory domain of YODA, which affects the activation of MITOGEN-ACTIVATED PROTEIN KINASE 3 (MPK3) and MITOGEN-ACTIVATED PROTEIN KINASE 6 (MPK6) kinases, inhibiting *SPCH* transcription. PHYTOCHROME INTERACTING FACTOR 4 (PIF4) can specifically bind to a regulatory sequence within the *SPCH* promoter and inhibit its expression. **(D)** ELONGATED HYPOCOTYL5 (HY5), activated by light, binds to the *STOMAGEN* promoter, promoting its activity, which translates into the inhibition of MAPK kinase activity. Inhibiting the activity of this kinase promotes *SPCH* expression. **(E)** In the presence of Jasmonic acid (JA) TFs from the MYC family (MYC2, MYC3 and MYC4) bind to the promoter of the *LOWER CELL DENSITY* (*LCD)* gene, which is one of the H_2_S synthesis enzymes, thus increasing its amount in the cell. The increased amount of H_2_S causes inhibition of *SPCH* expression. SI stands for Stomata Index. The illustration was created using BioRender (www.biorender.com).

In the ABA-related signal transduction pathways, the critical role play subclass III SnRK2 kinases, including SnRK2.2, SnRK2.3, and SnRK2.6 kinases, which are positive regulators via AREB/ABF through their phosphorylation (Fujita et al., 2013) Analysis of mutants in genes *SnRK2.2*, *SnRK2.3* and *SnRK2.6* showed an increase in SI in the *snrk2.2* and *snrk2.3* mutants, and even more significant increase in SI was observed in the *snrk2.2/snrk2.3* double mutant. Mutation in the *SnRK2.6* gene does not cause significant changes in SI, but its overexpression decreases SI. There was also no inhibitory effect of ABA on SI in the *snrk2.2/2.6/2.3* triple mutant, indicating that these kinases are involved in ABA-dependent inhibition of stomatal development. The expression pattern of genes encoding these kinases revealed that *SnRK2.2* and *SnRK2.3* were expressed in precursor cells, while *SnRK2.6* was expressed in mature guard cells. Using translational reporters for *SPCH* and *SnRK2.2* showed that they are co-expressed in precursor cells, proving the possibility of *SPCH* regulation by kinases. In addition, it was proved that artificially induced *SnRK2.6* at the early stages of stomatal development causes a decrease in SI, which proves its suppressor role in the early stages of stomatal development. The bimolecular fluorescent complementation (BiFC) assays between the SnRK2s and *SPCH* showed that all three kinases directly interact with *SPCH*. In addition, using phosphoproteomic studies, potential SnRK2 kinase target sites in *SPCH* promoters were identified. Potential target sites identified are within the mitogen-activated protein kinase (MAPK) target domain (MPKTD) at positions S240 and S271 (Yang et al., 2022) (**Figure 2A**). Another subclass of SNF1-related protein kinase 1 (SnRK1) kinases, which consists of subunits KIN10, KIN11, and two regulatory subunits β and γ in Arabidopsis are also involved in controlling stress reactions in plants (Kulik et al., 2011). KIN10 plays a role in the regulation of growth, development, and energy signaling, it has also recently been proven that it takes part in the autophagy processes in *A. thaliana* because its overexpression increases the production of autophagosomes (Chen et al., 2017). Recent studies have shown that *A. thaliana* KIN10 plays a role in stomatal development. Overexpression *KIN10* in plants growing in liquid MS medium with 1% sucrose increases SI compared to WT, while loss-of-function mutation causes a decrease in SI under the same conditions. The use of trehalose-6-phosphate (Tre6P), which is an inhibitor of KIN10, resulted in a decrease in SI, while the mutation in the *Trehalose-6-Phosphate Synthase1* (*TPS1*) encoding a key enzyme of Tre6P biosynthesis, resulted in a significant increase in SI, which confirms that KIN10 is a positive regulator of stomatal development. Protein–protein pull-down assays showed that KIN10 interacts with SPCH (**Figure 2B**) but does not affect MUTE and FAMA. These studies support the role of KIN10 in controlling stomatal development by interacting with SPCH (Han et al., 2020).

The *ERECTA* gene encodes a protein with a serine-threonine kinase domain belonging to the LRR-LRK family, which controls the transcription of genes essential for better tolerance to drought (Blair et al., 2015; Li et al., 2021). *ERECTA* controls the development and arrangement of stomata (Blair et al., 2015). Recently, a genetic pathway including HD-ZIP transcription factor EDT1/HDG11 ENHANCED DROUGHT TOLERANCE1/HOMEODOMAIN GLABROUS11 (EDT1/HDG11), E2Fa transcription factor and ERECTA were found to be responsible for better drought tolerance by increasing WUE and reducing stomatal density. The double mutant *edt1/er-105*, in which *EDT1/HDG1* is overexpressed while *ERECTA* is knockout, exhibited that stomata density is at a similar level as in the single *er-105* mutant, but stomatal density in a single *edt1* mutant being significantly lower. Stomatal density is negatively correlated with WUE and photosynthesis but positively correlated with transpiration rate (Guo et al., 2019). This demonstrates that knocking out the *ERECTA* gene blocks the function of EDT1/HDG1 in regulating stomatal density and WUE. It was also checked whether there are potential TF HD-ZIP binding sites within the *ERECTA* promoter. Two sequences were identified: AAATTAGT and TAATAATTA, for which Yeast one-hybrid assays showed that EDT1/HDG1 could bind to both sequences. These results were confirmed by ChIP-qPCR, which proves that EDT1/HDG1 can bind to the *ERECTA* promoter, activating transcription. Since cell size is positively correlated with the level of ploidy in *edt1* and *er-105* mutants, an attempt was made to identify nuclear factors involved in the process of cell cycle regulation that interacts with ERECTA. For this purpose, a two-hybrid yeast screen was used, where the ERECTA kinase domain was used as bait. The E2Fa protein that interacts with ERECTA has been identified. This protein regulates proliferation and endocycle in Arabidopsis (Magyar et al., 2012; Guo et al., 2019). Using *ERECTA* overexpression lines with the *E2F* knockout (35S::ERe2fa) showed that *E2Fa* affects *ERECTA*-dependent stomatal density. The level of cell ploidy was measured using flow cytometry, where it was significantly higher than in WT with overexpression of *ERECTA*, and more than half lower in the mutant *e2fa*. Using qRT-PCR, the relative expression levels of genes involved in the G1 to S phase transition and cell cycle progression of genes that are targets of E2F were checked. The obtained results allowed to conclude that *ERECTA* modulates the expression of genes related to the cell cycle through E2F, affecting the premature entry of cells into the endoreduplication cycle, resulting in the formation of stomata of larger sizes (Guo et al., 2019).

MicroRNAs are non-coding RNA molecules that are negative regulators of gene expression at the post-transcriptional level. It regulates many biological processes, including response to abiotic stress and tissue-specific development and stem cell differentiation (Zhao et al., 2007; Chen et al., 2012; Sun, 2012). In *H.vulgare*, expression of miRNA393 increases in drought conditions (Zhao et al., 2007). According to the latest research, miRNA393, through the auxin signalling pathway, may control stomata development in barley (Yuan et al., 2019). Studies on the expression pattern using the pMIR393a:GUS and pMIR393b:GUS reporter lines showed that miRNA393 is expressed, among others, in the stomata guard cells. To investigate the function of miRNA393, two transgenic *H. vulgare* lines were generated. Line 35S::MIR393b (*OE*) with overexpressing *miRNA393* and line 35S::MIM393 (*MIM*), in which miRNA393 expression was suppressed. Overexpression of miRNA393 increased stomatal density while reducing guard cell size, with the opposite phenotype observed in miRNA393 mutant plants. Using qRT-PCR, it was checked whether the mutation and overexpression of miRNA393 affected the expression of genes controlling stomatal development: *SPCH*, *MUTE*, and *FAMA*. Expression of *SPCH* and *MUTE* genes was increased in the *MIM* line, while it was decreased in the *OE* line. An increase in *FAMA* expression was observed in the *OE* line and a decrease in the *MIM* line. Level of expression of three genes *INDOLE-3-ACETIC ACID INDUCIBLE 12* (*IAA12)*, I*NDOLE-3-ACETIC ACID INDUCIBLE 12* (*IAA17)*, and *ABA Response Factor 5 (ARF5)* related to the auxin pathway and control of stomatal development was also checked. In both *OE* and *MIM* lines, the expression level of *IAA12* and *IAA17* was increased, with the opposite trend observed in the *ARF5* expression. Transgenic *H. vulgare* lines treated with drought showed that the *OE* line was much more sensitive to drought conditions, while the *MIM* line was much better at dealing with drought than the WT. In addition, the rate of water evaporation by transpiration in the *MIM* line was observed to be much lower than in the WT and *OE* (Yuan et al., 2019).

Transcription factors (TFs) play an essential role in the organism by controlling gene expression, thus impacting almost all developmental processes. TFs from the NAC family, which are characteristic of plants and involved in shoot apical meristem development, flower development, and cell division, also are engaged in biotic and abiotic stress regulation. It is also worth mentioning that the NAC family is one of the most numerous TF families in plants, e.g. 117 potential TFs from the NAC family were identified in *A. thaliana* and 151 in *Oryza sativa* (Hu et al., 2006; Nakashima et al., 2012; Shao et al., 2015). The expression of the *Z. mays* NAC family transcription factor *ZmNAC49* is at its highest levels in the leaves and stems of young plants. Treatment with Polyethylene Glycol (PEG6000) to mimic dehydration stress conditions has shown that expression is rapidly and significantly induced, suggesting a role for *ZmNAC49* in drought response. ZmNAC49 overexpressing line (OE)s: OE-ZmNAC49#2 and OE-ZmNAC49#4 were generated and subjected to drought stress. *ZmNAC49* overexpressing lines showed less leaf wilt compared to WT. In addition, transpiration and stomatal conductance in the OE line were lower than in the WT line. The density of stomata in OE lines was also 10% lower than WT. Additionally, stomatal precursor cells were observed, which were not present in the WT. It was also tested whether ZmNAC49 affects the expression of critical genes that control stomatal development. The expression of *ZmTMM*, *ZmSDD1* and *ZmFAMA* in the OE lines was higher, while *ZmMUTE* was decreased. No differences were observed in the expression of the *ZmSPCH* and *ZmSTOMAGEN* genes. Within the *ZmMUTE* promoter, the CACGTA sequence to which NAC family TFs specifically bind has been identified. The ChIP-qPRC assay using OE lines confirmed that ZmNAC49 can specifically bind to the *ZmMUTE* promoter. It was also checked how ZmNAC49 affects the expression of *ZmMUTE* under drought conditions. The OE and WT lines under optimal conditions showed that in the OE line the expression of *ZmNAC49* was higher and *ZmMUTE* was lower than WT. Drought treatment significantly increased *ZmNAC49* expression in both WT and OE lines and suppressed *ZmMUTE*. The obtained results confirmed the role of ZmNAC49 in the response of maize to drought stress, and as the reason for this better tolerance, they indicate the participation of ZmNAC49 in the suppression of *ZmMUTE* expression (Xiang et al., 2021).

During abiotic stresses, reactive oxygen species (ROS) are produced, such as superoxide radical, hydrogen peroxide (H_2_O_2_), or nitric oxide (NO), which are involved in defense mechanisms and act as signalling particles (Lim et al., 2015). Smaller amounts of ROS are formed as a side effect of the aerobic metabolism of plants. In addition to their signalling functions, which are important in the case of the stress response, they also cause DNA damage that is dangerous for the plant and damage to critical cellular proteins, which prevents them from making their original function (Huang et al., 2019). The ROS are also involved in stomatal closure. They are mainly produced by NADPH oxidases present in membranes. In *A. thaliana*, two major ABA-dependent isoforms of NADPH oxidases are present in the plasma membrane of the stomatal guard cells: AtRBOHF and AtRBOHD. The *atrbohF* mutant shows a reduced stomatal response when treated with ABA, with the *atrbohF* and *atrbohD* double mutant responding more strongly, but the *atrbohD* single mutant does not respond to ABA-related stomatal closure. ROS produced by NADPH cause the opening of Ca^2+^ channels, where the influx of calcium into the cells stimulates the production of reactive oxygen species in the cytosol by intracellular NADPH either directly by binding to them or indirectly by binding to kinases acting on these oxidases. Increased production of ROS affects the disruption of Ca^2+^ oscillations between cells, which contributes to apoplastic stomatal closure. The presence of ROS may change the activity of domains, e.g. in the OST1 kinase, and change the activity of K^+^ ion channels (Sierla et al., 2016; Ma and Bai, 2021).

## Effect of heat on stomatal development

The escalation in global temperatures has heighetened the interest of scientists in the subject of plant resistance to heat stress. By 2100, average annual temperatures will increase by about 0.3-4.8 degrees Celsius (Jagadish et al., 2021). Rising temperatures are becoming a significant limitation for the yield of cultivated cereals. While the oceans have absorbed 90% of the excess heat from greenhouse gases, land temperatures are rising faster than ocean surface temperatures. The 2021-22 assessment by the Intergovernmental Panel on Climate Change (IPCC) emphasizes that even a minor increase in global temperature results in significant and often severe local and regional impacts. This increase has notably heightened the probability of extreme weather and climate events. Furthermore, the planet is approaching vital ecological thresholds, previously only theorized (Tollefson, 2023). Under the influence of heat stress, the percentage of seed germination is significantly reduced and the percentage of photosynthesis efficiency decreases. In addition, during the reproduction period, the functions of the wallpaper cells are lost, and the anthers become dysplastic under the influence of elevated temperatures. Heat stress also inhibits the swelling of pollen grains and prevents the anthers from cracking, which results in a weaker release of pollen grains (Qu et al., 2013).

Heat Shock Factors (HSFs) are involved in heat avoidance by protecting the plant from the heat by activating and controlling the signal transduction network (Qu et al., 2013; Ohama et al., 2017). HSF1 ACTIVATOR 1 (HSF1A1a) is one of the primary regulators of the reaction to heat stress, while the main inducer of this reaction is HSF1 ACTIVATOR 2 (HSFA2) (Scharf et al., 2012; Qu et al., 2013). The genes encoding Heat Shock Proteins (HSPs) and ROS capture enzymes are targets of transcription factors that respond to heat stress. HSPs are involved in refolding proteins that have been changed conformationally due to high temperature and have lost the ability to perform their functions (Ohama et al., 2017). Acclimatization to high temperatures is also possible due to the activity of PHYTOCHROME INTERACTING FACTOR 4 (PIF4), a bHLH transcription factor (Proveniers and Van Zanten, 2013). PIF4 activity is regulated by various environmental factors: light, temperature, hormones (auxin, gibberellin), and the circadian clock at the transcriptional and post-transcriptional levels (Choi and Oh, 2016; Franklin et al., 2011). PIF4 acts as an integrator of environmental signals, mediating signal transduction between them and target signalling pathways, modulating signal transduction, which allows for the optimization of growth in thermal conditions (Choi and Oh, 2016). It has been shown that under heat stress conditions, the expression level of *PIF4* and the accumulation of its protein in the plant increases (Foreman et al., 2011). The role of the PIF4 protein in the context of stomatal development has recently been studied (Lau et al., 2018). Under high-temperature conditions, both PIF4 accumulation in the plant and suppression of *SPCH* expression occurred. Studies on *A.thaliana*, including wild-type (WT) and *PIF4* loss-of-function mutants (*pif4* and *pif4-2*), showed that the stomatal index (SI) at high temperatures (28°C) under long-day conditions (LD) and short-day (SD) conditions in WT are reduced, while in mutants they were not significantly changed, with a more pronounced decrease in SI observed in SD. PIF4-mediated responses are more noticeable in SD conditions (Yamashino et al., 2013; Lau et al., 2018). Analyses of the WT and *pif-4* mutant at low temperatures (12°C) showed that SI was not significantly different, supporting that the effect of PIF4 at low temperatures is not significant in stomatal development. In addition, using the *PIF4* expression reporter containing the GFP protein (PIF4pro:PIF4-GFP), it was proved that *PIF4* is expressed in stomatal cell precursors at high temperatures. Overexpression of *PIF4* under high-temperature conditions (28°C) decreased *SPCH* expression, while the *pif4* mutation resulted in a slight increase in *SPCH* expression compared to WT (Lau et al., 2018). The CANNTG motif has been identified in the *SPCH* promoter, the characteristic binding site for TF with bHLH family, to which PIF4 belongs (Amoutzias et al., 2008; Lau et al., 2018). Chromatin immunoprecipitation showed enrichment in promoter regions containing this motif in products encoded by the *PIF4* (**Figure 2C**) (Lau et al., 2018).

YODA signalosome is also involved in regulating the response to high temperatures. Central component involved in YODA signalosome is the Heat Shock Protein 90 (HSP90) chaperone, which interacts with YODA to modulate the phosphorylation of downstream targets. These targets include mitogen-activated protein kinases (MPK3 and MPK6), which control the expression of *SPCH* gene involved in stomatal formation and differentiation. Analysis of *HSP90* loss-of-function mutants *hsp90.1*, *hsp90.2*, and *hsp90RNAi* under optimal and heat stress conditions showed that the stomatal differentiation index decreased under both - optimal and heat conditions, confirming the vital role of HSP90 in the YODA signalosome (Samakovli, Tichá, and Šamaj 2020). The analysis of mutants in *YODA* gene revealed that it affects the differentiation of stomata. Heat-stressed WT *A.thaliana* plants and *yda-1* mutants that carried a nonsense mutation in the kinase domain showed that WT reduced SI under stress conditions. In contrast, the *yda-1* mutant exhibited stomatal clustering, indicating the involvement of *YODA* in the regulation of the stomata differentiation. Additionally, using HSP90 inhibitors 17-dimethylaminoethylamino-17-demethoxygeldanamycin (17-DMAG) and geldanamycin (GDA) it was checked whether HSP90 plays a role in the stomatal formation. It was shown that stomata clustering was promoted in WT under optimal conditions (22°C) by blocking HSP90 by inhibitors, while in *yda-1* mutants, stomatal density was reduced. The proSPCH:GUS marker lines were used in the *hsp90* and *hsp90RNAi* mutants to test whether HSP90 affected *SPCH* expression. Under control conditions, high activity of *SPCH* promoters was observed, while under heat stress, no activity of these promoters was observed. It was also checked whether using HSP90 inhibitors and mutations in the *HSP90* gene - *hsp90.1* and *hsp90RNAi- affect* symmetric and asymmetric cell division during stomata formation. Inhibition of *HSP90* activity under control conditions increased the number of cells like stomatal precursors. Under heat stress, WT plants showed increased stomatal precursors, while HSP90 knockout plants did not increase the number of stomatal cells in response to heat stress. Using yeast two-hybrid (Y2H), co-immunoprecipitation, and bimolecular fluorescence complementation (BiFC) assays, it was confirmed that HSP90 interacts with YODA, especially with its N-terminal regulatory domain (**Figure 2C**) (Samakovli et al., 2020b).

## Effect of light stress on stomata development

Light impacts on the opening of the stomata by activating receptors located on the surface of the guard cells. These receptors enhance the action of proton pumps (H^+^ATPase), so that H^+^ ions flow into the guard cells, causing the stomata open (Ou et al., 2014). Here, receptors that react to red light as (phytochromes) or blue light (cryptochrome or phototropins) are involved. These proteins undergo phosphorylation under the influence of blue light. Phosphorylated phototropins affect the activation of H^+^ATPases, which push H^+^ ions out of the cell while increasing the negative potential inside the cell, opening voltage-gated K^+^ channels. The influx of K^+^ ions into the cell reduces the osmotic potential, causing water to flow into the cell, thus opening the stomata. In *A.thaliana*, the MYB60 transcription factor is a positive regulator of stomatal opening in response to blue light. Its expression is specific to stomatal guard cells, and the knockout *atmyb60* mutation affects stomatal closure. Another TF is AtMYB61, a negative regulator of stomatal opening in response to light. Overexpression of this gene induces stomatal closure, while the *atmyb61* mutant is characterized by stomatal opening, its expression is also characteristic of stomatal guard cells (Chen et al., 2012; Ou et al., 2014). Recent studies on the *ENHANCED RESPONSE TO ABA 1* (*ERA1)* gene, which encodes the beta subunit of farnesyltransferase, have shown that it is involved in blue light-dependent stomatal opening (Cutler et al., 1996; Jalakas et al., 2017). The *era1-2* mutant has lower stomatal conductance than WT. *A. thaliana* double mutants were derived by crossing *era1-2* with *ost1-3* and *abi1-1*, whose phenotypes show high stomatal conductance. The analysis of the stomatal conductance of the double mutants showed that it is lower than the single mutants *ost1-3* and *abi1-1*. The influence of stomatal closing factors on the *era1 ost1* double mutant was investigated. Reactions in the double mutant were like in the single mutant *ost1*. Reactions of the *era1* mutant to light-conditioned stomatal opening were also checked. The stomatal opening was initially the same in WT and *era1.* However, after 60 minutes, stomatal conductance was higher in WT than in *era1*. Since stomatal opening in response to light is mainly driven by blue light, how blue light-driven stomata opening in WT and era1 works was examined. Measurement of stomatal conductance showed that light-driven stomatal opening is impaired in *era1*. This study revealed that *ERA1* is an important component in blue light-dependent stomatal opening and suggests a potential role for farnesylation in blue light-dependent stomatal opening processes (Jalakas et al., 2017).

Light also influences stomata development via ELONGATED HYPOCOTYL5 (HY5) (Wang et al., 2021). HY5 is a transcription factor of the bZIP family. In Arabidopsis, it acts as a growth regulator - it inhibits the growth of the hypocotyl and lateral roots, and its action is light-dependent (Gangappa and Botto, 2016; Bhatnagar et al., 2020). Recently, the role of HY5 in stomata development has been further investigated. *A. thaliana* plants with overexpression of the *HY5-OX* gene and its loss-of-function mutant *hy5-215* were analyzed. Overexpression of *HY5* resulted in an increase in both SI and SD in proportion to light intensity, while the *hy5-215* mutant had lower SD and SI compared to controls independent of light intensity. Counting the SLGC cells, it was found that the *hy5-215* mutant had a lower number of these cells compared to WT, while *HY5-OX* had a higher number of this type of cells than controls, which indicates that HY5 influences stomatal development at an early stage. Using a translational reporter for the *SPCH* gene (SPCHpro:SPCH-CFP), it was demonstrated that *SPCH* expression can be induced by light. Using the same reporter in the *hy5-51* mutant showed that the light-promoted expression of *SPCH* is at a lower level than in WT. Using a MAPK-inactive *SPCH* reporter (SPCHpro: SPCH2-4A-YFP) it was tested whether post-translational interactions with *SPCH* are involved in the control of this gene function. An accumulation of SPCH2-4A-YFP was observed in both darkness and light, suggesting that MAPK plays an important role in suppressing *SPCH* under dark conditions (**Figure 2D**). The expression of *STOMAGEN* - a gene that inhibits the action of MAPK is increased by about 20% in *HY5-OX* plants compared to WT, while in the *hy5-51* and *hy5-215* mutants, it is reduced by about 20%. Using translational reporter promoter-driven GUS reporter (STOMAGENpro:GUS), it was confirmed that *HY5* and *STOMAGEN* are expressed in the same cellular compartments, allowing HY5 to interact with *STOMAGEN*. *STOMAGEN* was also shown to function downstream of HY5 by contributing to the control of stomatal development. The amiR-stomagen knockdown mutant overexpressing *HY5 (HY5-OX)* showed suppression of stomatal production, i.e., the amiR-stomagen mutant suppressed the HY5-OX-expressed phenotype. Potential HY5 binding sites were checked within *STOMAGEN*. Within the *STOMAGEN* promoter, ACTG (Z-Box motif), a motif that HY5 recognizes, was found to be present, indicating the direct possibility of HY5 controlling *STOMAGEN*. An *in vitro* Electrophoretic Mobility Shift Assay (EMSA) test was performed, which confirmed the possibility of HY5 binding to *STOMAGEN* (Wang et al., 2021).

## The impact of salinity conditions on stomata patterning and functioning

Salt stress is estimated to affect up to 20% of arable land in the world (Negrão et al., 2017). It is connected with the excessive uptake of Na^+^ and Cl^-^ ions from the soil, which may impair the proper functioning of water management in the plant (Isayenkov and Maathuis, 2019; Negrão et al., 2017). During salt stress, the synthesis of ABA increases with a simultaneous decrease in the availability of K^+^ ions and the accumulation of H_2_O_2_. The roots are the main site of H2O2 production during salt stress, and it is formed because of the action of NADPH oxidases (Hedrich and Shabala, 2018). Then it is transported to the shoot, where it can affect the functioning of membrane transporters responsible for transporting K^+^ and Ca^2+^ ions by binding to cysteine residues. The presence of ABA under stress causes the opening of S-type anion channels: SLAC1 and SLAH3. Their phosphorylation is the result of the ABA signalling pathway, in which the cytosolic receptor bound to the ABA membrane PYR/PYL/RCAR, thereby inactivating ABA Insensitive 1 (ABI1), which was associated with *OST1*, conditioning its inactivity. Released from the control of ABI1, OST1 undergoes autophosphorylation, thereby transphosphorylating S-type anion channels, opening them, resulting in loss of turgor in the cell (Hedrich and Shabala, 2018). All these factors in themselves cause the stomata to close, and the presence of all of them at the same time only intensifies this process, which significantly inhibits the photosynthesis process and may limit the growth of biomass (Parihar et al., 2015; Hedrich and Shabala, 2018; Ghori et al., 2019).

In the case of disturbances in the osmotic balance, the water potential decreases (Parihar et al., 2015). To inhibit the negative impact of salt stress, the plant activates defense mechanisms, which include closing the stomata and inhibiting cell expansion (Isayenkov and Maathuis, 2019). Recent studies on the histone deacetylase HDA704 in *O.sativa* have shown that it determines a better tolerance to salt stress (Zhao et al., 2021) Histone deacetylases (HDAC) are enzymes that affect gene expression by removing the acetyl group from histones. Deacetylation causes the chromatin structure to become more compact, which prevents gene transcription (Ma et al., 2013; Liu et al., 2014).

Studies on HDA704 in *O. sativa* under salt and drought stress conditions showed that its expression was strongly induced by both salt and drought stress. Treatment of plants with ABA showed that *HDA704* expression was increased 3 hours after treatment, suggesting that HDA704 may be involved in the response to abiotic stresses and the response to ABA. The use of the reporter HDA704-EGFP proved that *HDA704* is expressed in the cell nucleus. Two overexpression lines of *HDA704* (OE) were generated: HDA704OX1 and HDA704OX14. Overexpressed lines showed a higher chlorophyll and carotenoid content under stress conditions, and during drought, the water content in their leaves was higher than in WT. These parameters were reduced compared to WT in generated knockout mutants HDA704RNAi2 and HDA704RNAi3 (KO). It was checked how overexpression and knockout affect stomatal opening. Under optimal water conditions, the OE line stomata have a wider aperture than the WT line, while in the KO line aperture of the stomata was narrower. In WT, the number of fully open stomata was higher than in the OE line but lower than in the OE line. It was also checked how drought affects the number of fully open stomata. In drought treated OE lines were observed to have wider stomata aperture than the WT, while KO lines aperture of stomata was lower. It has also been proven that overexpression of *HAD704* causes an increase in the expression of genes such as *ERECTA* or *SUB9*, which is a homolog of the Arabidopsis *SDD1* gene. *SDD1* is responsible for regulating stomata density, and its overexpression causes a decrease in density, the *sdd1* mutant shows an increased density. HDA704 has also been shown to interact with *Drought and Salt Tolerant (DTS)* and *Abscisic Acid-Insensitive Like2 (ABIL2)*, which are negative regulators of ABA signalling. Their expression levels were downregulated in the OE lines. Using Chromatin Immunoprecipitation (ChIP) it was demonstrated that the acetylation level of histones H3 and H4 in the promoters of the *ABIL2* and *DST* genes was downregulated in OE lines. Anti-HDA704 antibody and ChIP-PCR were used to check if it interacts with the *ABIL2* and *DST* promoters. The analysis showed an enrichment of the promoter of these genes in HDA704, which indicates that it affects the level of H3 and H4 acetylation. The H3 and H4 acetylation level of the *ABIL2* and *DST* genes in response to drought was also determined. The level of acetylation in *DST* H3 was decreased, while the level of H4 acetylation in *ABIL2* was increased, coinciding with a decrease in *DST* expression and an increase in *ABIL2* expression. The obtained results show that HDA704, through the acetylation of histones in the promoters of *ABIL2* and *DST* genes, affects their expression level and modulates the pattern of stomata distribution and tolerance to drought and salinity stress. (Huang et al., 2009; Li et al., 2015; Zhao et al., 2021).

## The impact of heavy metals on the development of stomata

In the case of heavy metal stress, the hydrogen sulfide (H_2_S) molecule plays a signalling role, combating the adverse effects of heavy metal stress by mitigating the associated oxidative stress (Luo et al., 2020; Arif et al., 2021; Liu et al., 2021). The presence of heavy metals determines the production of reactive oxygen species such as superoxide radical (O•−), hydrogen peroxide (H_2_O_2_), and hydroxyl radicals (·OH), among others, as a result of the Haber-Weiss reaction. Reactive oxygen species cause lipid peroxidation of unsaturated fatty acids, which affects the disruption of the cytoplasmic membrane (Luo et al., 2020). H_2_S, next to nitric oxide (NO) and carbon dioxide (CO_2_), is the third most important naturally occurring gaseous signalling molecule in plants. It can move freely between plant cells, and its level in the plant is kept constant by enzymes (Luo et al., 2020; Arif et al., 2021). Recent studies have shown that H_2_S regulates stomatal development, mediated by jasmonic acid (JA). It was shown that treating *A. thaliana* with methyl jasmonate (MeJA) resulted in a decrease in both the stomatal density and SI. Treatment with Hypotaurine (HT), an H2S scavenger, increased stomatal density and SI. Treatment with both MeJA and HT reverses the inhibitory effect of MeJA on stomatal development. A mutation in the *LOWER CELL DENSITY* (*LCD)* gene, which encodes one of the main enzymes that catalyze cysteine to H_2_S in *Arabidopsis*, reduced endogenous H_2_S levels by about half. The phenotype of this mutant was characterized by a higher stomatal density and SI than WT and was insensitive to MeJA treatment. Treatment of plants with NaHS, which is an exogenous H_2_S donor, resulted in a decrease in stomatal density and a decrease in SI, while HT treatment blocked the inhibitory effect of NaHS on stomatal development. In the *myc234* mutant, characterized by a defect in JA signalling and high stomatal density, treatment with NaHS decreased SI and stomatal density. In addition, JA has been shown to promote the synthesis of endogenous H*_2_* S by upregulating the expression of genes encoding H*_2_* S synthesis enzymes such as *LCD*, mediated by the transcription factors MYC2, MYC3 and MYC4 (**Figure 2E**). H_2_S regulates stomatal development by affecting to the suppression the *TMM*, *SPCH*, *MUTE*, and *FAMA* genes that control stomatal development. The above results suggest that H_2_S inhibits the expression of basic genes involved in stomatal development, and JA mediates this process by controlling the expression of enzymes involved in the synthesis of endogenous H_2_S (Deng et al., 2020).

## Conclusion remarks

As sedentary organisms, plants were forced to develop unique mechanisms that allowed them to survive unfavorable environmental conditions. The role of stomata in avoiding abiotic stresses has been known for a long time, but the molecular basis of the mechanisms involved is still being researched. One of the most severe stresses is drought and heat, because they affect the greatest loss of water from the plant. Nevertheless, other stresses such as light stress, heavy metal stress or salinity are also widely studied in the context of the functioning of stomata and their functioning in the context of better adaptation of plants to unfavorable conditions. This review highlighted the latest reports on stomata development and function under abiotic stresses. Recent research shows that genes whose expression change is specifically related to the appearance of some stressor can affect the pattern, structure and opening of the stomata. Morphological changes in the distribution and structure occur mainly through interaction with the regulatory sequences of basic genes related to stomatal development, such as *MUTE*, *SPCH*, *FAMA*. An important challenge in researching the impact of stresses on stomata is the possibility of translating the obtained results to other species. Currently, research is conducted mainly on *A. thaliana*, a dicotyledonous species. It is known that the development of stomata between monocots and dicots may differ in many respects. Therefore, identifying a given molecular mechanism determining stress resistance in dicots may differ significantly in monocots. Thus, it seems necessary to extend the research to monocot species. Due to the changing climate and progressive pollution, which is also associated with soil contamination and degradation, it is extremely important to search for regulators of response to abiotic stresses. Research on the behavior and development of stomata in the presence of stress seems extremely promising. Identifying new molecular mechanisms related to the stomatal response to stress may contribute to generating new, more resistant plant varieties.

## Author contributions

HM: Writing - review & editing, Visualization, Writing - original draft. AD: Writing - review & editing, Conceptualization, Funding acquisition, Project administration, Supervision.
